# Hoehn and Yahr Stage and Non-Motor Symptom Burden in Parkinson’s Disease: A Cross-Sectional Study in Peru

**DOI:** 10.3390/medicina62071355

**Published:** 2026-07-14

**Authors:** Ana L. Sobrino-Galindo, Miguel A. Arce-Huamani

**Affiliations:** Public Health, Surveillance and Applied Research, Programa Académico de Medicina Humana, Facultad de Ciencias de la Salud, Universidad Privada Norbert Wiener, Lima 15046, Peru; a2017201265@uwiener.edu.pe

**Keywords:** Parkinson’s disease, Hoehn and Yahr, MDS-UPDRS, non-motor symptoms

## Abstract

*Background and Objectives*: Parkinson’s disease is a multidimensional neurodegenerative disorder in which motor and non-motor manifestations frequently coexist. This study aimed to evaluate the association between Hoehn and Yahr stage and non-motor symptom burden among patients with Parkinson’s disease in Peru. *Materials and Methods*: An analytical cross-sectional study was conducted in 92 patients with Parkinson’s disease registered or followed at Hospital San Juan Bautista de Huaral, Lima region, Peru, during 2025. Non-motor symptom burden was assessed using MDS-UPDRS Part I and categorized as mild, moderate, or severe. Clinical stage was assessed using the Hoehn and Yahr scale and grouped as early, intermediate, or advanced. Crude and adjusted ordinal odds ratios (ORs) with 95% confidence intervals (CIs) were estimated using ordinal logistic regression. *Results*: The median age was 65.0 years, 67.4% were male, and the median disease duration was 6.0 years. Moderate and severe non-motor symptoms were observed in 58.7% and 23.9% of patients, respectively. In the adjusted model, intermediate stage (adjusted OR = 5.89; 95% CI: 1.83–18.98) and advanced stage (adjusted OR = 27.74; 95% CI: 6.74–114.18) were associated with greater non-motor symptom severity. *Conclusions*: Higher Hoehn and Yahr stage was independently associated with greater non-motor symptom severity. These findings support systematic non-motor assessment, particularly in intermediate and advanced stages.

## 1. Introduction

Parkinson’s disease is no longer regarded as a purely motor disorder, but as a multidimensional neurodegenerative syndrome in which motor impairment, non-motor symptoms, functional decline, and quality of life are closely linked [1,2]. In 2021, an estimated 11.77 million people were living with Parkinson’s disease worldwide, with age-standardized prevalence, incidence, and disability-adjusted life-year rates of 138.63, 15.63, and 89.59 per 100,000 population, respectively [3]. Among individuals aged 55 years and older, global Parkinson’s disease prevalence increased by 281%, incidence by 219%, and disability-adjusted life years by 165% between 1990 and 2021, highlighting the growing public health impact of population aging [4]. In Peru, recent population-based research in the central highlands has shown that Andean communities remain underrepresented in door-to-door Parkinson’s disease prevalence studies, despite regional variability in epidemiological estimates [5]. In a Peruvian outpatient cohort of 60 adults with Parkinson’s disease, anxiety, depression, and mild cognitive impairment were reported in 93.2%, 88.6%, and more than 60% of patients, respectively, underscoring the clinical relevance of non-motor manifestations in local neurological care [6]. Therefore, the clinical expression of Parkinson’s disease in Peru should be examined beyond motor staging alone.

From a pathophysiological perspective, Parkinson’s disease is characterized by progressive degeneration of dopaminergic neurons in the substantia nigra pars compacta and abnormal α-synuclein aggregation into Lewy-related pathology. However, the disease process extends beyond the nigrostriatal motor system. Mitochondrial dysfunction, oxidative stress, neuroinflammation, impaired protein clearance, autonomic involvement, and multisystem propagation of α-synuclein pathology contribute to a broader neurodegenerative process. This multisystem involvement is consistent with contemporary reviews describing Parkinson’s disease as a heterogeneous syndrome in which dopaminergic, non-dopaminergic, autonomic, inflammatory, and protein-aggregation pathways contribute to both motor and non-motor manifestations [7,8,9,10]. This biological complexity helps explain why non-motor symptoms may appear early and involve sleep, mood, cognition, autonomic function, gastrointestinal function, and sensory processing. Metabolic stressors may also be relevant. Recent evidence suggests that recurrent hypoglycemic episodes and glycemic variability may contribute to neurodegenerative vulnerability in parkinsonian syndromes through oxidative stress and impaired neuronal energy regulation [11]. Therefore, evaluating non-motor symptom burden across Hoehn and Yahr stages is clinically meaningful because it reflects a broader disease process rather than a purely motor staging problem.

Non-motor symptoms are frequent even in the earliest clinical stages of Parkinson’s disease and may become more disabling as disease severity increases [12,13]. A recent systematic review and consensus model of early Parkinson’s disease identified more than 340 symptoms and impacts across motor, cognitive, psychiatric, sleep, sensory, speech, digestive, urinary, sexual, autonomic, physical, and psychosocial domains [14]. In patients classified within Hoehn and Yahr stages 1–3, 84% had at least one non-motor symptom, with a mean Non-Motor Symptoms Scale score of 46.22 ± 22.10, showing that clinically meaningful non-motor burden may already be present before advanced motor disability [15]. A 2025 cross-sectional study reported at least one non-motor symptom in all participants, with sleep/fatigue symptoms in 95%, urinary symptoms in 79%, and gastrointestinal dysfunction in 76%; total NMSS scores were also correlated with disease severity and quality of life [16]. In South Asia, a 2025 systematic review and meta-analysis including 50 studies and 4931 participants confirmed a high pooled prevalence of non-motor symptoms, with nocturia emerging as the most frequent symptom among studies using validated tools [17]. Together, these findings indicate that non-motor symptoms are not secondary or marginal findings, but core manifestations of Parkinson’s disease across stages and settings. This interpretation is also supported by systematic reviews and meta-analyses showing that non-motor symptoms may precede diagnosis, accumulate across the disease course, and substantially affect quality of life, especially through neuropsychiatric, cognitive, sleep, autonomic, and fatigue-related domains [18,19,20]. Depression is particularly relevant within the neuropsychiatric domain, as systematic review evidence indicates that depressive symptoms are common in Parkinson’s disease and may contribute to disability, poorer quality of life, and greater clinical complexity [21].

The Hoehn and Yahr scale remains widely used to summarize motor stage, but motor staging alone may not fully capture the clinical burden experienced by patients. In a 2025 study of 230 consecutive patients, 69.1% reported at least one non-motor symptom among their three most troublesome complaints, and cognitive impairment was associated with higher odds of reporting non-motor symptoms among the top complaints [22]. In the COPPADIS cohort of 603 patients, a staging model that combined Hoehn and Yahr stage with non-motor symptom burden showed progressively worse quality of life and disability as both dimensions increased [23]. A multicenter Japanese cross-sectional study also supported the need to evaluate patient characteristics associated with non-motor symptoms rather than relying only on motor classification [24]. In a Swedish registry-based cost study, formal care costs were 3.8 times higher among observations with at least 10 non-motor symptoms, and hallucinations or delusions were associated with an 80–94% increase in total costs [25]. Therefore, assessing the association between Hoehn and Yahr stage and non-motor symptom burden in Peru may help determine whether motor progression reflects a broader clinical burden in this underrepresented setting.

This study aimed to evaluate the association between Hoehn and Yahr stage and non-motor symptom burden among patients with Parkinson’s disease in Peru.

## 2. Materials and Methods

### 2.1. Study Design and Setting

We conducted an analytical cross-sectional study among patients with Parkinson’s disease in Peru. The study was carried out at Hospital San Juan Bautista de Huaral, located in the Lima region, Peru, during 2025. The study was designed to evaluate whether clinical stage, assessed using the Hoehn and Yahr scale, was associated with the severity of non-motor symptoms measured using Part I of the Movement Disorder Society–Unified Parkinson’s Disease Rating Scale (MDS-UPDRS Part I).

### 2.2. Study Population, Sample Size, and Sampling

The source population consisted of 113 patients with Parkinson’s disease who were registered or followed at Hospital San Juan Bautista de Huaral during the study period. Because a census-based sampling strategy was used, no formal sample size calculation was performed. All potentially eligible patients identified in the institutional registry were assessed for inclusion.

After applying the predefined eligibility and exclusion criteria, the final analytical sample included 92 patients with Parkinson’s disease. Patients were excluded if they had died before data collection, could not be contacted, did not respond to the contact attempt, or had no available telephone number. Because several excluded patients lacked complete or verifiable clinical information, a formal comparison between included and excluded patients in terms of age, sex, disease duration, or disease stage could not be performed reliably. Therefore, the final sample represented accessible patients who met the eligibility criteria and had complete information for the main study variables.

### 2.3. Eligibility Criteria

Patients were eligible if they were adults with a diagnosis of Parkinson’s disease established by a neurologist according to the Movement Disorder Society Clinical Diagnostic Criteria for Parkinson’s disease [26]. These criteria define parkinsonism as bradykinesia combined with either rest tremor or rigidity, followed by the clinical assessment of supportive criteria, red flags, and absolute exclusion criteria. Eligible patients also had to be registered or followed at Hospital San Juan Bautista de Huaral during 2025 and have available clinical information for disease staging using the Hoehn and Yahr scale and for non-motor symptom assessment using MDS-UPDRS Part I.

Patients were excluded if they had died before data collection, could not be reached by telephone, did not respond to the contact attempt, or had incomplete information for the main exposure or outcome variables. Patients who were unable or unwilling to provide the required information were not included in the final analytical dataset.

These exclusion criteria were applied to ensure that all included participants had reliable and complete information for both the main exposure and outcome variables. However, they may have influenced the final analytical sample. In particular, excluding patients who had died before data collection or who could not be contacted may have reduced the representation of patients with more severe disease, greater functional impairment, or poorer access to follow-up. Therefore, the final sample should be interpreted as a group of accessible patients with complete clinical and assessment data, rather than as the full institutional registry of patients with Parkinson’s disease.

### 2.4. Variables and Definitions

The main outcome variable was non-motor symptom severity, assessed using MDS-UPDRS Part I. This section evaluates non-motor experiences of daily living in patients with Parkinson’s disease, including cognitive, behavioral, mood, sleep, autonomic, sensory, and fatigue-related symptoms. The total MDS-UPDRS Part I score was categorized into three ordinal severity levels using previously proposed cut-off points for MDS-UPDRS severity classification: mild non-motor symptoms (≤10 points), moderate non-motor symptoms (11–21 points), and severe non-motor symptoms (≥22 points) [27].

The main explanatory variable was clinical stage according to the Hoehn and Yahr scale. This scale classifies Parkinson’s disease progression from stage 1 to stage 5 based on laterality of symptoms, postural instability, disability, and functional dependence. For the main analyses, Hoehn and Yahr stages were grouped as early stage (stages 1–2), intermediate stage (stage 3), and advanced stage (stages 4–5).

Covariates included age, sex, and disease duration. Age was measured in years and analyzed descriptively and as a continuous variable in regression models using 10-year increments. Sex was recorded as female or male. Disease duration was calculated as the difference between the year of evaluation and the year of Parkinson’s disease diagnosis and was analyzed descriptively and in regression models using 5-year increments. Levodopa equivalent daily dose was not included as a study variable because detailed antiparkinsonian medication doses were not consistently available in the clinical records. Similarly, dopaminergic treatment status and the motor state at the time of assessment (“on” or “off” state) were not systematically documented. In routine clinical practice, dopaminergic treatment is individualized according to clinical response, disease stage, tolerability, and motor complications, as reflected in the Peruvian clinical practice guideline for Parkinson’s disease [28].

### 2.5. Data Collection Procedures

Data were collected from clinical records and structured patient assessment. Sociodemographic and clinical information, including age, sex, year of diagnosis, and disease duration, was obtained from available clinical information and verified during patient contact when necessary.

Clinical staging was performed using the Hoehn and Yahr scale, and non-motor symptom burden was assessed using MDS-UPDRS Part I, which evaluates non-motor experiences of daily living. To reduce inter-rater variability, both Hoehn and Yahr staging and MDS-UPDRS Part I assessment were independently reviewed by two trained evaluators. Any discrepancies were discussed and resolved by consensus before final database entry. The collected information was then entered into a coded database to preserve confidentiality. Before analysis, the database was reviewed to identify missing values, inconsistencies, and implausible entries. Only patients with complete information for Hoehn and Yahr stage, MDS-UPDRS Part I score, and relevant covariates were retained in the final analytical dataset.

### 2.6. Statistical Analysis

Categorical variables were summarized using absolute and relative frequencies. Numerical variables were summarized using medians and interquartile ranges because normal distribution was not assumed. Baseline characteristics included age, sex, disease duration, Hoehn and Yahr stage, grouped Hoehn and Yahr stage, MDS-UPDRS Part I score, and non-motor symptom severity.

Bivariate analyses were performed to evaluate the association between non-motor symptom severity and clinical characteristics. Age and disease duration were compared across non-motor symptom severity categories using the Kruskal–Wallis test. Sex was compared using Pearson’s chi-square test or Fisher’s exact test, as appropriate. The association between grouped Hoehn and Yahr stage and non-motor symptom severity was evaluated using the Fisher–Freeman–Halton exact test because some expected cell counts were low. A two-sided *p* value < 0.05 was considered statistically significant.

To estimate the magnitude of the association between Hoehn and Yahr stage and non-motor symptom severity, crude and adjusted ordinal logistic regression models were fitted. The dependent variable was non-motor symptom severity, ordered as mild, moderate, and severe. The analysis focused on the overall MDS-UPDRS Part I score and its ordinal severity categories. Item-level or domain-specific analyses of individual non-motor symptoms were not performed because individual MDS-UPDRS Part I item scores were not available in the analytical dataset, which contained only the total score and severity category. Therefore, the study evaluated global non-motor symptom burden in relation to Hoehn and Yahr stage. The main independent variable was grouped Hoehn and Yahr stage, with early stage as the reference category. Ordinal odds ratios (ORs) with 95% confidence intervals (CIs) were estimated to express the odds of being in a higher non-motor symptom severity category. The adjusted model included grouped Hoehn and Yahr stage, age, sex, and disease duration.

Collinearity among the independent variables included in the adjusted model was assessed using tolerance values and variance inflation factors (VIFs). Problematic collinearity was defined as VIF ≥ 5 or tolerance ≤ 0.20.

Additionally, the association between Hoehn and Yahr stage and the continuous MDS-UPDRS Part I score was explored graphically using boxplots. Spearman’s correlation coefficient was calculated to assess the monotonic relationship between Hoehn and Yahr stage and non-motor symptom burden. Differences in MDS-UPDRS Part I scores across Hoehn and Yahr stages were evaluated using the Kruskal–Wallis test.

All statistical analyses were performed using IBM SPSS Statistics, version 24.0 (IBM Corp., Armonk, NY, USA). Statistical significance was set at *p* < 0.05.

### 2.7. Ethical Considerations

All participants or their appropriate informants received information about the study objectives, procedures, voluntary nature of participation, and confidentiality safeguards before data collection. Informed consent was obtained according to the approved protocol. The study involved minimal risk because no experimental intervention was performed, and information was obtained through clinical assessment and review of existing clinical data.

Confidentiality was maintained throughout the study. Each participant was assigned a coded identifier, and no directly identifying personal information was included in the analytical database. Access to the database was restricted to the research team, and all findings were reported in aggregate form to prevent individual identification.

## 3. Results

Table 1 presents the baseline characteristics of the study population. A total of 92 patients with Parkinson’s disease were included. The median age was 65.0 years (IQR: 58.8–73.3), and most participants were male (67.4%). The median disease duration was 6.0 years (IQR: 3.0–8.0), with 45.7% of patients having a disease duration of ≤5 years. Regarding clinical staging, Hoehn and Yahr stage 3 was the most frequent individual stage (34.8%), followed by stage 2 (26.1%) and stage 4 (18.5%). When stages were grouped, 42.4% of patients were classified as early stage, 34.8% as intermediate stage, and 22.8% as advanced stage. The median MDS-UPDRS Part I score was 17.0 points (IQR: 13.8–21.0). Overall, 58.7% of patients had moderate non-motor symptom severity, and 23.9% had severe non-motor symptoms.

Table 2 shows the bivariate associations between non-motor symptom severity and clinical characteristics. Median age increased from 62.5 years in the mild group to 71.0 years in the severe group, although this difference was not statistically significant (*p* = 0.255). Sex distribution was also similar across severity categories (*p* = 0.631). Disease duration showed a slight increase across non-motor symptom severity categories, but this association was not statistically significant (*p* = 0.313). In contrast, grouped Hoehn and Yahr stage was strongly associated with non-motor symptom severity (*p* < 0.001). Among patients with mild non-motor symptoms, 87.5% were in early stages and none were in advanced stages. Conversely, among patients with severe non-motor symptoms, 54.5% were in advanced stages, while only 13.6% were in early stages.

Table 3 presents the crude and adjusted ordinal logistic regression models for non-motor symptom severity. In the crude analysis, patients in Hoehn and Yahr stage 3 had higher odds of being in a more severe non-motor symptom category compared with those in stages 1–2 (crude OR = 5.79; 95% CI: 1.86–18.02; *p* = 0.002). This association was stronger among patients in stages 4–5 (crude OR = 26.02; 95% CI: 6.83–99.16; *p* < 0.001). After adjustment for age, sex, and disease duration, the association remained statistically significant. Patients in stage 3 had nearly six-fold higher odds of greater non-motor symptom severity (adjusted OR = 5.89; 95% CI: 1.83–18.98; *p* = 0.003), while those in stages 4–5 had markedly higher odds (adjusted OR = 27.74; 95% CI: 6.74–114.18; *p* < 0.001). Age, sex, and disease duration were not independently associated with non-motor symptom severity in the adjusted model.

**Figure 1:**
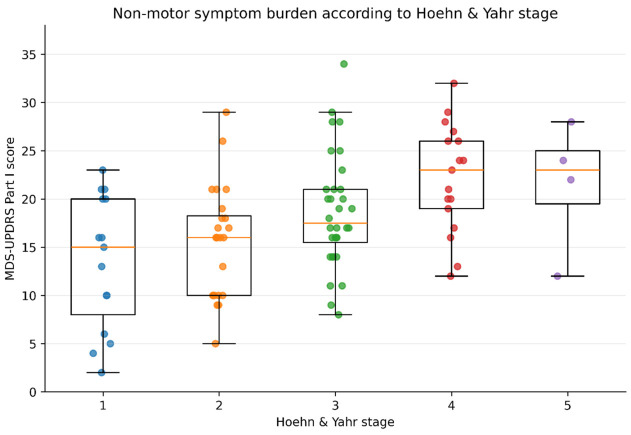
Non-motor symptom burden according to Hoehn & Yahr stage. Boxplots show the distribution of MDS-UPDRS Part I scores across Hoehn & Yahr stages, with individual observations overlaid. A progressive increase in non-motor symptom burden was observed across higher stages. Spearman’s rho = 0.43; *p* < 0.001, Kruskal–Wallis *p* = 0.002.

Figure 1 supports the pattern observed in the regression models. MDS-UPDRS Part I scores increased progressively across higher Hoehn and Yahr stages. This trend was statistically significant, with a moderate positive correlation between Hoehn and Yahr stage and non-motor symptom burden (Spearman’s rho = 0.43; *p* < 0.001). Differences in MDS-UPDRS Part I scores across Hoehn and Yahr stages were also significant according to the Kruskal–Wallis test (*p* = 0.002).

## 4. Discussion

In this cross-sectional study of Peruvian patients with Parkinson’s disease, higher Hoehn and Yahr stages were independently associated with greater non-motor symptom severity. The association followed a clear dose-response pattern: compared with patients in stages 1–2, those in stage 3 had nearly six-fold higher odds of being in a more severe non-motor symptom category, whereas patients in stages 4–5 had almost twenty-eight-fold higher odds after adjustment for age, sex, and disease duration.

The novelty of this study lies in showing, in a Peruvian clinical setting, that a simple and widely used motor staging scale is strongly associated with the severity of non-motor experiences of daily living measured using MDS-UPDRS Part I. This is clinically relevant because Latin American evidence on the relationship between motor stage and non-motor burden remains limited, and non-motor symptoms are often underrecognized in routine care. Our findings suggest that Hoehn and Yahr staging may help identify patients who require more systematic non-motor assessment, especially in intermediate and advanced stages. However, the moderate correlation between Hoehn and Yahr stage and MDS-UPDRS Part I also indicates that motor stage and non-motor burden are related but not interchangeable constructs. Therefore, Hoehn and Yahr staging should not replace direct non-motor symptom evaluation, but may serve as a practical clinical signal for more comprehensive assessment.

The clinical profile of our cohort was broadly comparable to that of large international Parkinson’s disease samples. In our study, patients had a median age of 65 years, most were male, and the median disease duration was 6 years. These characteristics are similar to those reported in the Parkinson’s Disease Non-Motor International Longitudinal Study, in which van Wamelen et al. [29] described a mean age of 65.9 years, mean disease duration of 6.3 years, and 64.2% male participants. This similarity suggests that, although our cohort was recruited from a Peruvian neurological setting, it was not substantially younger, more advanced, or more sex-skewed than major international cohorts. However, unlike multicenter studies with broad geographic representation, our sample reflects real-world patients from a single Latin American clinical setting, where access to specialized neurological care and timing of diagnosis may differ. Therefore, the comparability in age and disease duration supports the external plausibility of our findings, while the local setting adds contextual relevance for underrepresented health systems.

The high non-motor burden observed in our study is also consistent with contemporary evidence showing that non-motor symptoms are highly frequent and clinically meaningful in Parkinson’s disease. In our cohort, 82.6% of patients had moderate or severe non-motor symptoms, and the median MDS-UPDRS Part I score was 17 points. Similarly, Ahsan et al. [16] found that all patients had at least one non-motor symptom, with sleep/fatigue affecting 95%, urinary symptoms 79%, and gastrointestinal dysfunction 76%; total NMSS scores were also correlated with disease severity and quality of life. Martinez-Martin et al. [30] likewise showed that non-motor symptoms, including nocturia, fatigue, and sialorrhea, were frequent and that total NMSS correlated more strongly with PDQ-39 quality-of-life scores than motor severity. Together, these findings support the interpretation that non-motor symptoms are not secondary or marginal manifestations, but central components of the clinical burden of Parkinson’s disease.

The distribution of non-motor severity across Hoehn and Yahr stages followed a clinically coherent gradient. Among patients with mild non-motor symptoms, 87.5% were in early stages, whereas among those with severe non-motor symptoms, 54.5% were in advanced stages. This pattern is consistent with the findings of Skorvanek et al. [31], who showed that MDS-UPDRS scores across all four parts increased significantly with each Hoehn and Yahr stage, suggesting that motor staging is accompanied by worsening multidimensional disease burden. Ou et al. [32] also reported longitudinal progression of non-motor symptoms in early Parkinson’s disease, with the mean number of symptoms increasing from 7.5 to 9.5 over 3 years and higher baseline non-motor burden predicting faster motor progression. Together, these findings reinforce the biological and clinical plausibility of our results: as motor disability advances, non-motor dysfunction also tends to accumulate, probably reflecting broader neurodegenerative involvement beyond nigrostriatal dopaminergic pathways. Biologically, this interpretation is compatible with the concept that non-motor symptoms arise from distributed neurodegenerative involvement beyond dopaminergic motor circuits, including cortical, limbic, brainstem, autonomic, and peripheral pathways. This broader disease model helps explain why cognitive, psychiatric, sleep, autonomic, sensory, and fatigue-related symptoms may progress alongside motor disability rather than appearing as isolated late complications [9,33,34].

The strongest finding in our study was the independent association between Hoehn and Yahr stage and greater non-motor symptom severity. After adjustment, stage 3 was associated with markedly higher odds of greater non-motor severity, and stages 4–5 showed an even stronger association. This finding is aligned with the COPPADIS analysis by Kulisevsky et al. [23], which proposed combining Hoehn and Yahr motor stage with NMSS-based non-motor symptom burden because motor staging alone may not fully capture disability or quality of life. In that study, worse quality of life and greater disability were observed at higher combined motor–non-motor stages. Our findings support this conceptual approach: Hoehn and Yahr staging remains clinically informative, but its interpretation is strengthened when non-motor burden is measured directly.

Age, sex, and disease duration were not independently associated with greater non-motor severity in our adjusted model. This differs from some previous studies in which age, disease duration, or longitudinal progression were related to non-motor symptom burden. For example, Ou et al. [32] found that the number of non-motor symptoms was associated with age and motor severity, whereas van Wamelen et al. [29] reported significant differences in NMSS scores according to disease-duration groups, particularly between patients with disease duration below and above 5 years. Gustafsson et al. [25] also found that the number of non-motor symptoms increased with disease duration. In our cohort, collinearity diagnostics did not indicate problematic collinearity among the independent variables included in the adjusted model, with VIF values ranging from 1.04 to 1.37 and tolerance values ranging from 0.73 to 0.96. Therefore, the absence of independent associations for age and disease duration is unlikely to be explained by collinearity. A more plausible explanation is that Hoehn and Yahr stage captured part of the accumulated disease severity otherwise reflected by age or disease duration. In addition, disease duration showed limited variability, with an interquartile range of 3–8 years, which may have reduced the ability to detect an independent effect. Therefore, these findings should be interpreted cautiously and not as evidence that age or disease duration are clinically irrelevant to non-motor symptom burden. Sex-related differences in Parkinson’s disease may also influence clinical expression, progression, and non-motor manifestations; however, our study was not powered to explore sex-specific patterns in detail [35].

The moderate positive correlation between Hoehn and Yahr stage and MDS-UPDRS Part I further supports the multidimensional progression of Parkinson’s disease. In our study, higher Hoehn and Yahr stage correlated with higher non-motor symptom burden (rho = 0.43; *p* < 0.001). Skorvanek et al. [31] provide a relevant benchmark, showing in 3206 patients that MDS-UPDRS Parts I, II, III, and IV increased significantly across Hoehn and Yahr stages. This supports the use of MDS-UPDRS Part I as a clinically meaningful non-motor complement to motor staging. Nevertheless, the correlation in our study was moderate rather than strong, suggesting that motor stage and non-motor burden are related but not interchangeable constructs. Therefore, relying only on Hoehn and Yahr stage could underestimate clinically important non-motor manifestations in some patients.

This study also provides regionally relevant evidence from Peru, where Parkinson’s disease research remains less represented than European, North American, and Asian cohorts. Previous Peruvian evidence by Cosentino et al. [36] showed that non-motor symptoms were highly frequent among local Parkinson’s disease outpatients, with at least one symptom present in 99.3% of patients and a mean total NMSQuest score of 12.41. More recent Peruvian clinical data have also reported high frequencies of neuropsychiatric symptoms, pain, and cognitive impairment in outpatient Parkinson’s disease populations. Compared with these studies, our work adds an analytical focus on the association between motor stage and non-motor severity using Hoehn and Yahr staging and MDS-UPDRS Part I. This is relevant because locally generated evidence can help guide clinical screening priorities in settings where specialized movement-disorder services may be limited.

From a clinical perspective, our findings support systematic screening for non-motor symptoms, particularly in patients at intermediate and advanced Hoehn and Yahr stages. This recommendation is consistent with evidence showing that non-motor symptoms have consequences beyond clinical discomfort. Gustafsson et al. [25] reported that non-motor symptoms were present in 74% of Swedish registry observations, with a mean of 6.9 symptoms, and that formal care costs were 3.8 times higher among observations with at least 10 non-motor symptoms. Hallucinations and/or delusions were also associated with an 80–94% increase in total costs. These data suggest that identifying non-motor burden is important not only for symptom control and quality of life, but also for health-system planning. In this context, our results support the integration of MDS-UPDRS Part I into routine evaluation of Peruvian patients with Parkinson’s disease. This clinical recommendation is supported by recent evidence showing that non-motor symptoms are associated with substantial quality-of-life impairment, care needs, and cost burden. Because available treatment options remain heterogeneous and domain-specific, structured screening may help identify patients who require targeted multidisciplinary care rather than relying only on motor-stage assessment [37,38,39].

Overall, this study contributes to the Parkinson’s disease literature by showing that higher motor stage is strongly associated with greater non-motor symptom severity in a Peruvian clinical sample. This supports the concept that Parkinson’s disease progression should not be interpreted only through motor impairment, because patients in intermediate and advanced stages may also carry a substantial non-motor burden. Although the results should be generalized cautiously because they come from a single clinical setting, they may be relevant to similar neurology outpatient populations in resource-limited or middle-income settings where Hoehn and Yahr staging remains widely used and structured non-motor assessment is not always routine.

### Strengths and Limitations

The main strength of this study is its focus on a clinically relevant and underexplored question in a Peruvian Parkinson’s disease population: whether motor stage is associated with non-motor symptom severity. In addition, the combined use of Hoehn and Yahr staging and MDS-UPDRS Part I provides a practical framework for evaluating both motor progression and non-motor burden in routine clinical settings. The independent review of clinical staging and non-motor symptom assessment by two trained evaluators also helped reduce potential inter-rater variability. The ordinal regression model also allowed us to estimate the odds of belonging to a higher non-motor symptom severity category while adjusting for key demographic and clinical variables.

However, these findings should be interpreted in light of several important limitations. First, the cross-sectional design does not allow temporal or causal inference between motor progression and non-motor symptom burden. Therefore, the observed association should be interpreted as a clinical correlation rather than evidence that advancing Hoehn and Yahr stage directly causes greater non-motor severity. Second, the modest sample size may have limited the statistical power to detect independent associations with age, sex, or disease duration and may have contributed to wide confidence intervals in the ordinal regression models. Although collinearity diagnostics did not indicate problematic collinearity, the relatively narrow variability in disease duration may still have limited the ability to detect its independent contribution. Third, the exclusion criteria may have introduced selection bias, because patients who had died before data collection, could not be reached by telephone, did not respond to contact attempts, or had incomplete information were not included. Because complete demographic and clinical information was not uniformly available for excluded patients, we could not reliably compare included and excluded patients. As a result, patients with more advanced disease, greater disability, poorer social support, or limited access to follow-up may have been underrepresented.

Other clinical limitations should also be considered. Levodopa equivalent daily dose could not be reported because detailed antiparkinsonian medication doses were not consistently available in the clinical records. In addition, dopaminergic treatment status and the motor state at the time of assessment (“on” or “off” state) were not systematically documented. Therefore, residual confounding by antiparkinsonian treatment intensity or medication state cannot be ruled out. Parkinson’s disease motor subtype was not evaluated, although patients with postural instability and gait difficulty phenotypes may have a higher non-motor symptom burden than those with tremor-dominant phenotypes [40]. Therefore, the possibility that motor subtype partly influenced the observed association between Hoehn and Yahr stage and non-motor symptom severity cannot be ruled out. In addition, item-level or domain-specific analyses of MDS-UPDRS Part I could not be performed because individual item scores were not available in the analytical dataset. Therefore, we could not determine whether specific non-motor domains, such as cognitive, mood, sleep, autonomic, sensory, or fatigue-related symptoms, were more strongly associated with Hoehn and Yahr stage than others. Finally, because this was a single-center clinical study, the findings may not be fully generalizable to community-based patients, patients treated outside neurological care, or those without reliable access to follow-up.

## 5. Conclusions

In conclusion, higher Hoehn and Yahr stage was independently associated with greater non-motor symptom severity among patients with Parkinson’s disease in Peru. Patients in stage 3 and stages 4–5 had substantially higher odds of belonging to a more severe non-motor symptom category than those in stages 1–2, while age, sex, and disease duration were not independently associated after adjustment. The positive correlation between Hoehn and Yahr stage and MDS-UPDRS Part I further supports the coexistence of advancing motor disability and increasing non-motor burden. These findings reinforce the need to incorporate systematic non-motor symptom assessment into routine Parkinson’s disease care, particularly among patients in intermediate and advanced motor stages. In clinical practice, Hoehn and Yahr staging should be complemented by structured non-motor evaluation to improve symptom recognition, guide multidisciplinary management, and address the broader clinical burden of Parkinson’s disease.

## Figures and Tables

**Table 1:** Baseline characteristics of patients with Parkinson’s disease.

Variable	Category	n (%) or Median (IQR)
**Sociodemographic characteristics**		
**Age, years**	Overall	65.0 (58.8–73.3)
**Age group**	41–64 years	40 (43.5)
	65–74 years	30 (32.6)
	75–84 years	17 (18.5)
	≥85 years	5 (5.4)
**Sex**	Female	30 (32.6)
	Male	62 (67.4)
**Clinical characteristics**		
**Disease duration, years**	Overall	6.0 (3.0–8.0)
**Disease duration category**	≤5 years	42 (45.7)
	6–10 years	36 (39.1)
	11–15 years	7 (7.6)
	>15 years	7 (7.6)
**Disease staging and non-motor burden**		
**Hoehn & Yahr stage**	Stage 1	15 (16.3)
	Stage 2	24 (26.1)
	Stage 3	32 (34.8)
	Stage 4	17 (18.5)
	Stage 5	4 (4.3)
**Hoehn & Yahr grouped stage**	Early, stages 1–2	39 (42.4)
	Intermediate, stage 3	32 (34.8)
	Advanced, stages 4–5	21 (22.8)
**MDS-UPDRS Part I score**	Overall	17.0 (13.8–21.0)
**Non-motor symptom severity**	Mild	16 (17.4)
	Moderate	54 (58.7)
	Severe	22 (23.9)

Note. Values are presented as n (%) for categorical variables and median (interquartile range) for numerical variables. Disease duration was calculated as 2025 minus year of diagnosis. MDS-UPDRS, Movement Disorder Society–Unified Parkinson’s Disease Rating Scale; IQR, interquartile range. Bold text in the first column indicates section headings and main variable names.

**Table 2:** Bivariate associations between non-motor symptom severity and clinical characteristics.

Variable	Category	Mild, n (%) or Median (IQR)	Moderate, n (%) or Median (IQR)	Severe, n (%) or Median (IQR)	*p*-Value
**Age, years**	Overall	62.5 (60.5–65.5)	66.5 (57.3–73.8)	71.0 (60.0–77.5)	0.255
**Sex**	Female	5 (31.2)	16 (29.6)	9 (40.9)	0.631
	Male	11 (68.8)	38 (70.4)	13 (59.1)	
**Disease duration, years**	Overall	4.5 (3.0–5.3)	6.0 (3.3–8.8)	6.0 (3.5–10.3)	0.313
**Hoehn & Yahr grouped stage**	Early, stages 1–2	14 (87.5)	22 (40.7)	3 (13.6)	<0.001
	Intermediate, stage 3	2 (12.5)	23 (42.6)	7 (31.8)	
	Advanced, stages 4–5	0 (0.0)	9 (16.7)	12 (54.5)	

Note. Percentages are calculated within each non-motor symptom severity category. *p*-values were calculated using chi-square tests, Fisher–Freeman–Halton exact tests or Kruskal–Wallis tests, as appropriate. MDS-UPDRS Part I severity was categorized as mild (≤10 points), moderate (11–21 points), and severe (≥22 points). Bold text in the first column indicates the main variable names.

**Table 3:** Crude and adjusted ordinal odds ratios for non-motor symptom severity.

Variable	Category	Crude Ordinal OR (95% CI)	*p*-Value	Adjusted Ordinal OR (95% CI)	*p*-Value
**Hoehn & Yahr grouped stage**	Early, stages 1–2	Reference		Reference	
	Intermediate, stage 3	5.79 (1.86–18.02)	0.002	5.89 (1.83–18.98)	0.003
	Advanced, stages 4–5	26.02 (6.83–99.16)	<0.001	27.74 (6.74–114.18)	<0.001
**Age**	Per 10-year increase	1.39 (0.97–2.01)	0.076	0.99 (0.66–1.47)	0.943
**Sex**	Female	Reference		Reference	
	Male	0.72 (0.30–1.72)	0.462	0.92 (0.37–2.30)	0.865
**Disease duration**	Per 5-year increase	1.07 (0.72–1.58)	0.731	0.90 (0.62–1.31)	0.570

Note. OR odds ratio; CI, confidence interval; MDS-UPDRS, Movement Disorder Society–Unified Parkinson’s Disease Rating Scale. Ordinal ORs estimate the odds of being in a higher non-motor symptom severity category. Crude estimates were obtained from bivariate ordinal logistic regression models. Adjusted estimates were obtained from a multivariable ordinal logistic regression model including Hoehn & Yahr grouped stage, age, sex, and disease duration. Bold text in the first column indicates the main variable names.

## Data Availability

The de-identified dataset and analytic syntax are available from the corresponding author upon reasonable request, subject to institutional approval and applicable ethical restrictions.

## Abbreviations

The following abbreviations are used in this manuscript:

CI	Confidence interval
CIEIC-UPNW	Institutional Committee of Ethics and Scientific Integrity of Universidad Privada Norbert Wiener
DALY	Disability-adjusted life year
H&Y	Hoehn and Yahr
IQR	Interquartile range
MDS-NMS	Movement Disorder Society Non-Motor Rating Scale
MDS-UPDRS	Movement Disorder Society–Unified Parkinson’s Disease Rating Scale
MDS-UPDRS Part I	Non-motor experiences of daily living section of the Movement Disorder Society–Unified Parkinson’s Disease Rating Scale
NMS	Non-motor symptoms
NMSS	Non-Motor Symptoms Scale
NMSQuest	Non-Motor Symptoms Questionnaire
OR	Odds ratio
PD	Parkinson’s disease
PDQ-39	39-item Parkinson’s Disease Questionnaire
